# Changes in brain network dynamics during functional/dissociative seizures: An exploratory pilot study on EEG microstates

**DOI:** 10.1016/j.ebr.2025.100809

**Published:** 2025-07-23

**Authors:** Domantė Kučikienė, Johannes Jungilligens, Stefan Wolking, Yvonne Weber, Jörg Wellmer, Stoyan Popkirov

**Affiliations:** a Department of Epileptology and Neurology, RWTH Aachen University, Aachen, Germany; b Department of Neurology, University Hospital Knappschaft Kliniken Bochum, Bochum, Germany; c Ruhr-Epileptology, Department of Neurology, Knappschaft Kliniken University Hospital Bochum, Bochum, Germany; d Department of Neurology and Centre for Translational Neuro- and Behavioural Sciences (C-TNBS), University Hospital Essen, Essen, Germany

**Keywords:** Functional/dissociative seizures, Psychogenic nonepileptic seizures, Functional neurological disorder, EEG microstates

## Abstract

The pathophysiology of functional/dissociative seizures (FDS), also known as psychogenic nonepileptic seizures, remains incompletely understood. Current theories suggest that ictal changes in self-awareness and behavioural control are likely related to arousal-mediated disruptions of brain network dynamics, but direct electrophysiological evidence is scarce. In a proof-of-concept, the second of its kind pilot study, we explored ictal changes in EEG microstates – quasi-stable patterns of electrical activity of 50–70 ms duration that represent fundamental building blocks of large-scale brain network dynamics. Across a sample of 13 FDS patients, four microstates yielded a high mean global explained variance of 76.2 % and qualitatively resembled the well-established “canonical” microstate map topographies A-D. Repeated measure analysis of variance did not reveal any significant differences in contribution, occurrence or global field power of microstates between baseline and ictal recordings. Microstate duration, however, was significantly different between baseline and seizure recordings with shorter durations of microstates in FDS (p = 0.007). This was most pronounced for microstate D (Cohen’s *d* = 0.75) with the change being significant in an exploratory post hoc paired *t*-test (p = 0.044). Since microstate D is thought to reflect frontoparietal network activity, the findings of this pilot study can be interpreted as supportive of current theories of arousal-mediated disruptions of network activity that reduce cognitive and behavioural control during FDS.

## Highlights

• A significantly shorter microstate duration during FDS was found.
• This difference was most pronounced for microstate D.
• Microstate D has been previously shown to correspond to the frontoparietal network.
• This could indicate the disruptions of frontoparietal network activity during FDS.

## 1 Introduction

Functional/dissociative seizures (FDS), also known as psychogenic nonepileptic seizures, are sudden episodes of altered perceptual awareness featuring convulsive, collapsing or stuporous movement patterns [1]. They combine psychoform dissociation in response to dysregulated arousal with a disinhibition of involuntary and usually stereotyped motor behaviours shaped by innate defensive automatisms and socioculturally meaningful movements. The psychological mechanisms of FDS occurrence have been studied in depth [2], and different forms of psychotherapy have been developed and tested [3]. The neural correlates of FDS, on the other hand, remain largely unknown, not least due to the technical difficulty of orchestrating reliable neurophysiological measurements during seizures. Until recently, meaningful ictal analysis of brain function using surface and intracranial EEG, functional MRI or single-photon emission computed tomography (SPECT) have been performed on only a handful of patients, revealing subtle changes in brain networks involved in self-perception, emotion regulation, and motor control. [[4], [5], [6], [7], [8]]. However, these findings remain largely heterogeneous and divergent, highlighting the need for further research towards a coherent neurobiological understanding of FDS.

Current theories regarding the neurophysiology of FDS broadly converge on a momentary disruption of global brain network activity as a candidate mechanism [[9], [10], [11]]. More specifically, recent neuroimaging studies have proposed that alterations of brainstem-mediated arousal and its effect on cortical network activity could underlie the phenomenological changes seen during FDS [12,13].

Contrary to epileptic seizures, FDS are not associated with visually recognizable EEG changes. However, advanced quantitative EEG analysis might help detect subtle changes in brain activity during FDS. One such approach that has recently been developed is EEG microstate analysis [14]. Microstates are very short (50–70 ms), stable patterns of EEG electric field topography that reflect synchronized activity in large-scale cortical networks. Instead of focusing on focal changes in different regions, they approach the global brain activity at every time point. These transient configurations have been linked to distinct cognitive and physiological processes, such as sensory processing, executive control, and self-referential thought [14,15]. Previously, resting-state EEG analysis revealed statistically significant differences in microstates in lower frequency bands between patients with FDS compared to patients with epilepsy [16]. In a very recent study, changes in microstate parameters during FDS in comparison with baseline activity were demonstrated for the first time, suggesting that this is a promising methodology for examining underlying ictal neurophysiology [17]. Although offering interesting and promising results, microstate research in FDS still remains scarce. In this pilot study, we aimed to compare EEG microstate characteristics between baseline and ictal EEG recordings of FDS patients from a clinical setting.

## 2 Materials and methods

### 2.1 Subjects

This study utilized a retrospective convenience sample of previously identified patients with FDS established by semiological analysis combined with ictal video-EEG [18]. All subjects were inpatients of the Department of Neurology of the Knappschaft Kliniken University Hospital Bochum, Germany, which incorporates a level 4 epilepsy centre. For the final analysis, 13 seizures from 13 subjects (10 women, 3 men, mean age 33.9 ± 18.3 years) with sufficient duration of artefact-free ictal and baseline EEG recording during which patients had their eyes closed continually were identified. Further clinical information on the patient sample is presented in the supplementary Table 1.

### 2.2 EEG recording, segmentation and preprocessing

All included subjects had available EEG data for baseline and ictal conditions during the same recording session, that was free of major muscle or movement artefacts, and during which the eyes were closed. In cases, when patients briefly opened and closed their eyes during the baseline or ictal recording, these segments were removed. This was important, since eye opening significantly alters microstate topography [19]. A subset of eight subjects had a clearly identifiable onset of FDS and sufficient recording with eyes closed preceding it, allowing for an additional “preictal” EEG-recording. 19 EEG electrodes were placed according to the international 10–20 system. The mean recording duration was 3.68 ± 2.70 min.

The pre-processing of the raw EEG data was performed using the MATLAB (R2022b)-based EEGLAB toolkit (v2021.1) [20]. First, we applied a band-pass filter from 1 to 30 Hz. The reference channel was set to average. We inspected every recording visually and removed pronounced artifacts. Then, independent component analysis was performed. We classified and removed artefacts using previously published algorithms for automated multiple artifact rejection [21] as well as for ocular artefacts [22]. Finally, we performed an additional manual qualitative and quantitative check.

### 2.3 Microstate analysis

The EEG data was analysed using an EEGLAB microstate-analysis plug-in [23] for MATLAB. We followed standard procedure for EEG microstate analysis [15,16]. First, we calculated microstates in each recording: the global field power (GFP) parameter was used to define electric EEG activity at each timepoint, including the data from all EEG electrodes. The topographic maps of GFP-peaks were then clustered using the modified *k*-means algorithm. The polarity was ignored. Clustering into four microstates was chosen, as it showed a high global explained variance (GEV), while also qualitatively resembling the canonical microstate map topographies. Clustering into more than four microstates did not increase the GEV in a significant way. After identifying microstates in each recording, four mean microstate maps – MS-A, MS-B, MS-C and MS-D across all EEGs were calculated. Those were then backfitted to every recording and the quantitative calculations were performed. The statistical analysis included standard microstate parameters: the duration of each microstate in milliseconds, the contribution of each microstate to the entire data, the occurrence of each microstate per second, and mean GFP in μV. We additionally analysed microstate transition parameters, i.e., how frequently one microstate transitioned to a distinct other microstate (e.g., MS-A to MS-B, MS-A to MS-C etc.). Due to the different length of EEG-recordings, all statistical parameters were also corrected for the duration of recording by assigning a weight to each parameter depending on the length of the analysed EEG segment.

### 2.4 Statistical analysis

We used repeated measures ANOVA (rmANOVA) for the analysis of the microstate parameters between baseline and ictal conditions. We used standard microstate parameters (duration, contribution, occurrence and GFP) as dependent variables and condition (baseline or ictal) and microstate (MS-A to MS-D) as independent variables. First, we checked whether independent variables alone had any significant effect on the dependent variable. Afterwards we analysed the combined effect of both independent variables on the dependent variable in order to reveal any microstate-specific changes. To analyse the transition probabilities of microstates, we used transition probability in percent as a dependent variable, condition (baseline or ictal) and microstate transition pairs (e.g., MS-A to MS-B, MS-A to MS-C etc.) as independent variables and followed the previously described process. Paired t-tests were used for exploratory post hoc comparisons. Effect sizes (Cohen’s d) were calculated by dividing differences in means by pooled standard deviations. Significance level was set at <0.05.

### 2.5 Ethics

This study involves human participants and was approved by the ethic committee of the Medical Faculty, Ruhr University Bochum (Reg. No. 23-7849-BR). This is a retrospective study based on data originally collected for diagnostic purpose. The study complied with the Code of Ethics of the World Medical Association (Declaration of Helsinki).

## 3 Results

### 3.1 General microstate characteristics

**Fig. 1:**
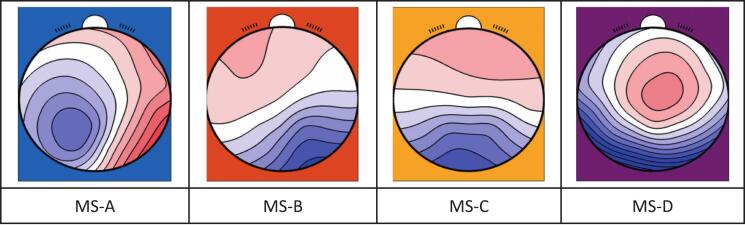
Microstate topographies.

The mean GEV of the four microstates was 76.2 % (SD 6.2 %), which is comparable with other studies [14]. The topography of microstates A-D (Fig. 1) was similar to the previously described four “canonical” microstates [24], a quantitative topographical analysis can be found at Table 4 (Supplementary material).

### 3.2 Baseline vs. ictal microstates

**Fig. 2:**
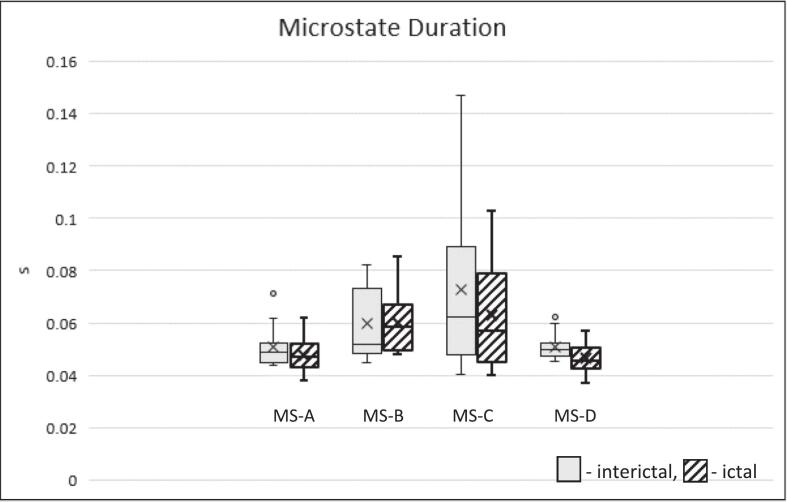
Microstate duration in seconds.

We found a shorter duration of all microstates in the ictal condition, compared to the baseline condition (MS-A_bl: 49.9 ± 7.5 ms; MS-B_bl: 60.9 ± 13.1 ms; MS-C_bl: 71.2 ± 27.5 ms; MS-D_bl: 50.1 ± 4.1 ms vs. MS-A_ic: 47.4 ± 6.5 ms; MS-B_ic: 59.9 ± 10.9 ms; MS-C_ic: 62.7 ± 19.1 ms; MS-D_ic: 46.2 ± 6.1 ms, F = 8.798; p = 0.007) Fig. 1, Fig. 2. In the rmANOVA analysis, this difference was not specific to any particular microstate (F = 1.521, p = 0.242). This analysis precludes examining the durations of individual microstates. However, this rests on the presumption, that durations of different microstates are interdependent. To test whether different microstates are indeed interrelated, we examined whether they were statistically independent. Establishing independence would justify post hoc comparisons of individual microstate durations between conditions, whereas interdependence would preclude such analyses. To this end, we performed correlation analysis between microstate durations across conditions as well as between the respective differences between baseline and ictal conditions. While differences between baseline and ictal conditions were not interrelated (all p > 0.05, see supplementary Table 2), overall durations of microstates A-C showed significant correlations (supplementary Table 3). Only microstate D duration showed no significant correlation with the other states. Thus, an exploratory paired *t*-test was performed comparing microstate D duration between baseline and ictal conditions and showed a significant reduction during seizures (t = 2.25; df = 12; p = 0.044). The difference corresponded to an effect size of Cohen *d* = 0.75, which was notably larger than changes seen in the other microstates (0.07–0.36).

Rm-ANOVA did not reveal any significant differences in contribution, occurrence or GFP of microstates between baseline and ictal recordings. Transition probabilities of different microstates were also not significantly different between conditions.

### 3.3 Baseline vs. preictal vs. ictal

A sub-set analysis of the eight subjects, who had EEG data for three discrete conditions (baseline, preictal and ictal) did not reveal any significant differences in microstate duration, contribution, occurrence, GFP or transition probabilities.

## 4 Discussion

An acute disruption of brain network activity is currently considered the likeliest neural correlate of FDS [11]. Ictal activity in FDS is generally rarely captured in EEG and does not show any specific changes in comparison to baseline EEG. EEG microstate analysis, reflecting global network changes, could offer a novel insight into the alterations of network dynamics during FDS. In an attempt to capture that, we performed EEG microstate analysis on a highly selected sample of FDS patients. In our exploratory pilot study, we found shorter durations of microstates during FDS compared to baseline values. The temporal characteristics of EEG microstates are essential for maintaining network properties, which support flexible and adaptive brain activity [25]. Changes in microstate durations have been previously found in neurological disorders such as Lewy body dementia, where an increase in duration of across microstates is correlated with the severity of cognitive fluctuations [15]. Shorter microstate durations could similarly weaken system stability, potentially leading to the temporary loss of cognitive adaptability and flexibility seen in dissociative states. In an EEG microstate study of hypnotic conditions, deep hypnosis was associated with significant changes in the duration of three microstates, supporting the claim that changes in durations are associated with changes in cognitive flexibility [26].

Post hoc exploratory testing of our data revealed that the shortening of microstate durations was most pronounced (largest effect size) and statistically significant in microstate D. Source localisation analyses and EEG-fMRI studies have previously suggested that the canonical microstate D likely corresponds to activity of the frontoparietal network and is primarily associated with executive processes [14,[27], [28], [29]]. In a large study that probed subjective experiences at rest using the Amsterdam Resting-State Questionnaire (covering ten dimensions of mind wandering), the duration of microstate D was inversely correlated with the “Self” dimension, which subsumes the three items “I thought about my feelings/my behaviour/myself” [30,31]. A shorter duration of microstate D would thus indicate a dissociation from external environment and a relative preponderance of inwardly directed mentation. In the above mentioned study on hypnotic states, microstate D had markedly shorter mean duration during deep hypnosis than during rest with similar effect size to that found in our study [26]. In the only two available case studies of incidental intracranial EEG recording during FDS (in patients with comorbid epilepsy), ictal changes were seen in parietal cortex connectivity, in line with potential disruptions of frontoparietal network activity [6,8].

In a recent study on ictal changes of microstate characteristics in patients with FDS, which employed similar methodology to our study, only microstate C showed significant reductions in duration compared to resting state baseline [17]. Of note, microstate C duration during resting state EEG was longer in patients compared to healthy control participants, highlighting its potential role in FDS pathology. Further studies will elucidate which differences in methodology likely explain the discrepancy of findings. However, the commonality – a reduction in microstate duration – has to be highlighted as a novel potential neural correlate of acute dissociation during FDS.

Taken together, reduced EEG microstate durations during FDS could be an indicator of ictal network instability, particularly regarding frontoparietal network activity. Further research in this area could open a door towards non-behavioural treatment paradigms. For example, psychological effects of various non-invasive brain stimulation protocols are associated with specific changes in EEG microstate dynamics [[32], [33], [34]]. Microstate-Neurofeedback based on microstate D contribution has also been successfully implemented in healthy volunteers and clinical population [35,36].

The retrospective study design and the small sample are the major limitations of our study. Excluding patients with major muscle or movement artefacts also limits the generalizability of our findings – the neural correlates of hypokinetic FDS might be different from those of the hyperkinetic type. Furthermore, in akinetic seizures the exact onset is sometimes difficult to discern, as outward seizure manifestations are subtle, potentially introducing methodological inaccuracies.

In conclusion, our analysis of EEG microstate dynamics during FDS reveals a reduction in microstate durations, most pronounced in microstate D, which is thought to reflect frontoparietal network activity. This finding supports current theories of arousal-mediated disruptions of network activity that reduce cognitive and behavioural control during FDS [12,13,37]. A microstate-based biomarker of FDS, once independently replicated, could pave the way for novel diagnostic approaches and targeted therapeutic strategies, potentially including neurofeedback or non-invasive neurostimulation.

## CRediT authorship contribution statement

**Domantė Kučikienė:** Writing – review & editing, Writing – original draft, Software, Methodology, Investigation, Formal analysis, Conceptualization. **Johannes Jungilligens:** Methodology, Formal analysis, Conceptualization. **Stefan Wolking:** Writing – review & editing, Methodology, Conceptualization. **Yvonne Weber:** Writing – review & editing, Supervision. **Jörg Wellmer:** Writing – review & editing, Supervision. **Stoyan Popkirov:** Writing – review & editing, Writing – original draft, Methodology, Funding acquisition, Data curation, Conceptualization.

## Declaration of competing interest

The authors declare the following financial interests/personal relationships which may be considered as potential competing interests: SP is supported by a BMBF Advanced Clinician Scientist Programme UMEA^2^ (01EO2104).
