# Supplementary material for: Changes in brain network dynamics during functional/dissociative seizures: An exploratory pilot study on EEG microstates
Source: Epilepsy Behav Rep. 2025 Jul 23;31:100809. doi: 10.1016/j.ebr.2025.100809 (PMC12318286; doi:10.1016/j.ebr.2025.100809)
Supplement: Supplementary Data 1 [file mmc1.docx]

**SUPPLEMENTARY MATERIAL**

**Supplementary Table 1.** Characteristics of patients.

| **Age** | **Gender** | **Seizure frequency** | **Seizure semiology** | **Medication** |
| --- | --- | --- | --- | --- |
| 29 | F | yearly | initially tingling sensation and feeling of weakness in both legs, then symmetrical high-frequency low-amplitude trembling and shaking of both legs | none |
| 34 | F | daily | feeling of tension in the legs, headache, irregular breathing, shoulders pulled up, whole body tensing up, head reclined, tearful crying, pressed voice, feeling of "deep sadness", sustained responsiveness | levetiracetam |
| 19 | F | monthly | irregular involuntary jerks and thrashing movements of the left extremities, some jerks with right arm, incomplete opisthotonos | none |
| 19 | F | weekly | heat and tingling sensations of the body, then slight trembling and then back-and-forth movement of arms and legs, incomplete opisthotonos, sustained responsivity throughout | none |
| 20 | M | unclear | varying convulsions of the entire body with stuttering and various tingling and painful sensations, no change in alertness or responsiveness | lamotrigine |
| 59 | F | unclear | nodding and trembling of the right hand and then left leg, intensification and generalisation of convulsions to affect whole body, then loss of verbal responsivity and incomplete memory, cued recall of questions; later reports feeling "far away" during seizure | levetiracetam, promethazine, pregabalin, venlafaxine, metformin, sitagliptin, bisoprolol, ramipril, hydrochlorothiazide, simvastatin, pantoprazole |
| 17 | F | unclear | irregular tremor of the right hand, feeling of warmth, accelerated breathing, tingling sensation in the feet | none |
| 59 | M | unclear | minor motor restlessness with intermittend jerks of the head and both legs, alert throughout |  |
| 66 | F | daily | after hyperventilation exercise prolonged loss of responsiveness for several minutes | none |
| 27 | F | unclear | sudden lifting of the left arm, looks around the room with wide open eyes, no adequate response to address, left hand held with fingers splayed, slight turning of the head towards person addressing her, the upper body is jerked backwards several times and the whole body is tensed (opisthotonos-like movement), hands held in claw position | none |
| 55 | F | weekly | tingling sensation of the lower lip and right hand, turns head to the right and it falls limply to the right, spreads her left arm supporting on the back of the chair, eyes closed, unresponsive to speech, slowed response to pain stimuli, tingling sensation of the whole body, slight toning of the arms and legs with recurrent slight jerks of the legs upward and adduction, fingers in claw posture, reports 'funny head feeling', nausea and dizziness ictally, breathes faster and with difficulty, somewhat fatigued afterwards | lamotrigine, citalopram, etoricoxib |
| 19 | M | unclear | high-frequency back-and-forth movement of the left arm, then feeling of tightness and pain in the chest, responsiveness unimpaired | none |
| 18 | F | unclear | loss of responsiveness, eyelid flutter, irregular opisthotonos, grimacing, upper body rocking motions, dystonic posturing oft both arms/hands as well as jaw, sobbing, fluctuating course with brief intermittent responsiveness | none |

**Supplementary Table 2.** Correlations between baseline-to-ictal differences of microstates A-B.

|  |  | **Delta_1** | **Delta_2** | **Delta_3** | **Delta_4** |
| --- | --- | --- | --- | --- | --- |
| **Delta_1** | **Spearman's Rho** | — |  |  |  |
|  | **df** | — |  |  |  |
|  | **p-Wert** | — |  |  |  |
| **Delta_2** | **Spearman's Rho** | 0.341 | — |  |  |
|  | **df** | 11 | — |  |  |
|  | **p-Wert** | 0.254 | — |  |  |
| **Delta_3** | **Spearman's Rho** | 0.412 | -0.052 | — |  |
|  | **df** | 11 | 11 | — |  |
|  | **p-Wert** | 0.163 | 0.865 | — |  |
| **Delta_4** | **Spearman's Rho** | 0.516 | 0.168 | 0.082 | — |
|  | **df** | 11 | 11 | 11 | — |
|  | **p-Wert** | 0.074 | 0.584 | 0.792 | — |

**Supplementary Table 3.** Correlations between durations of Microstates A-D, baseline and ictal recordings combined.

|  | | | | | |
| --- | --- | --- | --- | --- | --- |
|  |  | **Duration_1_combined** | **Duration_2_combined** | **Duration_3_combined** | **Duration_4_combined** |
| **Duration_1_combined** | **Spearman's Rho** | — |  |  |  |
|  | **df** | — |  |  |  |
|  | **p-Wert** | — |  |  |  |
| **Duration_2_combined** | **Spearman's Rho** | 0.575* | — |  |  |
|  | **df** | 11 | — |  |  |
|  | **p-Wert** | 0.040 | — |  |  |
| **Duration_3_combined** | **Spearman's Rho** | 0.008 | 0.709** | — |  |
|  | **df** | 11 | 11 | — |  |
|  | **p-Wert** | 0.979 | 0.009 | — |  |
| **Duration_4_combined** | **Spearman's Rho** | 0.283 | 0.231 | 0.269 | — |
|  | **df** | 11 | 11 | 11 | — |
|  | **p-Wert** | 0.348 | 0.448 | 0.373 | — |
| Note: * p < .05, ** p < .01, *** p < .001 | | | | | |

**Supplementary Table 4.** Correlations between Microstates A-D Topographies and
“Canonical” microstate maps published by T. Koenig in 2002

| Kucikiene et al., 2025  T. Koenig et al., 2002 | A | B | C | D |
| --- | --- | --- | --- | --- |
| A | R=0,433  P<0,001 |  |  |  |
| B |  | R=0.886  P<0.001 |  |  |
| C |  |  | R=0.865  P<0.001 |  |
| D |  |  |  | R=0.796  P<0.001 |

R= correlation coefficient, p = p-value
